# Acquired Amegakaryocytic Thrombocytopenia With Evolution Toward Aplastic Anemia Treated With Thrombopoietin Receptor Agonists: A Case Report

**DOI:** 10.1155/carm/6274311

**Published:** 2026-05-15

**Authors:** Faramarz Jabbari-Zadeh, Mujtaba M. Basharat, Benjamin Chin-Yee, Chai W. Phua, Selay Lam, Anargyros Xenocostas, Eric To, Cyrus C. Hsia

**Affiliations:** ^1^ Department of Medicine, Schulich School of Medicine & Dentistry, London Health Sciences Centre, Western University, London, Ontario, Canada, uwo.ca; ^2^ Schulich School of Medicine & Dentistry, Western University, London, Ontario, Canada, uwo.ca; ^3^ Department of Medicine, Division of Hematology, Schulich School of Medicine & Dentistry, London Health Sciences Centre, Western University, London, Ontario, Canada, uwo.ca; ^4^ Department of Pathology and Laboratory Hematology, London Health Sciences Centre, Western University, London, Ontario, Canada, lhsc.on.ca

## Abstract

Amegakaryocytic thrombocytopenia is a rare hematological disorder that is characterized by absent or near‐absent megakaryocytes without evidence of other hematological abnormalities. The disease can either be congenital or acquired. Given that less than a hundred cases of acquired amegakaryocytic thrombocytopenia (AAMT) have been reported in the literature, its pathophysiology and treatment remain poorly understood. Various treatments have been proposed for AAMT, including glucocorticoids and other immunosuppressive agents, such as cyclosporine, antithymocyte globulin, and rituximab. To date, there are limited data on the role of thrombopoietin receptor agonists (TPO‐RAs) for the treatment of AAMT. We present the case of a patient who initially presented with ecchymoses, purpura, and petechiae and was ultimately diagnosed with AAMT. He was treated with eltrombopag and had an increase in platelets with decreased bleeding after approximately 6 months of eltrombopag therapy. He did not experience any side effects from eltrombopag or require any platelet transfusions during follow‐up. Treatment overlapped with cyclosporine and prednisone, but these were discontinued due to adverse effects. Our case demonstrates that TPO‐RAs can be effective novel therapeutic agents and improve patient outcomes for this rare condition.

## 1. Introduction

Megakaryocytes (MKs) are the hematopoietic precursor to platelets found in the bone marrow [1]. Amegakaryocytic thrombocytopenia is a rare hematological disorder that is characterized by absent or near‐absent MKs without additional hematological abnormalities [2]. The disease can be congenital, which manifests as very severe cases of thrombocytopenia at birth and can potentially progress to bone marrow failure. Conversely, amegakaryocytic thrombocytopenia can also be acquired, which generally manifests later in life, usually presenting with bleeding symptoms [2].

Given that less than a hundred cases of acquired amegakaryocytic thrombocytopenia (AAMT) have been reported in the literature, its specific pathophysiology and treatment options are not well understood. AAMT is often a heterogenous disorder and can be secondary to autoimmune disease, lymphoproliferative disorders, viral infections, environmental toxins, or due to a stem cell defect [3]. Various treatments have been proposed for AAMT, including glucocorticoids and other immunosuppressive agents, such as cyclosporine, antithymocyte globulin (ATG; a special antibody used to deplete T lymphocytes), and monoclonal antibodies such as rituximab [4].

There is, however, a notable lack of studies examining the efficacy of thrombopoietin receptor agonists (TPO‐RAs) for the treatment of AAMT. Thrombopoietin is a growth factor that is necessary for MK development. TPO‐RAs activate the same receptor as endogenous thrombopoietin and result in similar effects [5]. Examples of TPO‐RAs include eltrombopag, avatrombopag, and romiplostim [6]. Specifically, eltrombopag is used to treat various etiologies of thrombocytopenia, including chronic immune thrombocytopenia, thrombocytopenia secondary to hepatitis C virus, aplastic anemia (AA), and myelodysplastic syndromes [7, 8]. Aside from activating the TPO receptor, eltrombopag also chelates iron, which stimulates both hematopoiesis at the stem cell level and megakaryopoiesis [6]. Dosing usually ranges from 12.5–150 mg daily and is titrated based on platelet response [4, 8]. Adverse effects include headache, myalgias, anemia, gastrointestinal (GI) upset, hepatotoxicity, cataracts, and thrombosis [9, 10]. In this manuscript, we present the case of a patient with AAMT treated with eltrombopag who ultimately had an increase in platelets and decreased bleeding after approximately 6 months of therapy.

## 2. Case Description

An 84‐year‐old gentleman presented initially with fatigue, purpura, ecchymoses, and petechiae. He was referred for thrombocytopenia and macrocytic anemia found on subsequent investigations. Medical history included cardiovascular disease, atrial fibrillation, hypertension, and dyslipidemia requiring edoxaban, digoxin, perindopril, indapamide, allopurinol, and rosuvastatin. He had no history of alcohol consumption. On physical examination, there was no apparent adenopathy or hepatosplenomegaly.

Investigations identified a persistent thrombocytopenia for two years, and at the time of assessment, his platelets were 29 × 10^9^/L (reference range: 50–400), hemoglobin 105 g/L (reference range: 135–170), mean cell volume 113 fL (reference range: 80–100), and leukocytes 4.8 × 10^9^/L (reference range: 4.0–10.0). His creatinine was 113 μmol/L (reference range: 62–120), which was at his baseline. His international normalized ratio was 1.2 (reference range: 0.9–1.1) and activated partial thromboplastin time 32 s (reference range: 19–29). His gamma glutamyl transferase (GGT) was elevated at 147 U/L (reference range: < 60), but all of his other liver enzymes were within the reference range. His serum protein electrophoresis (SPEP) demonstrated a monoclonal IgG lambda peak at 2.1 g/L. His total serum protein and immunoglobulin levels were within the reference range.

His vitamin B12 level was 577 pmol/L (reference range: 145–569). Additionally, his lactate dehydrogenase was increased at 455 U/L (reference range: < 225) with undetectably low haptoglobin and normal total bilirubin. His reticulocyte count was 80 × 10^9^/L (reference range: 10–100). His hepatitis B serology was unremarkable. A direct antiglobulin test was negative. His peripheral blood smear only showed evidence of macrocytosis without any schistocytes. Peripheral blood myeloid next‐generation sequencing (NGS) panel did not demonstrate any underlying myeloid mutations. Peripheral blood flow cytometry revealed a small paroxysmal nocturnal hemoglobinuria (PNH) clone of undetermined significance, glycosylphosphatidylinositol (GPI) deficient white blood cells (neutrophils 0.79% and monocytes 2.05%), and total GPI deficient red blood cells 0.47%.

**FIGURE 1:**
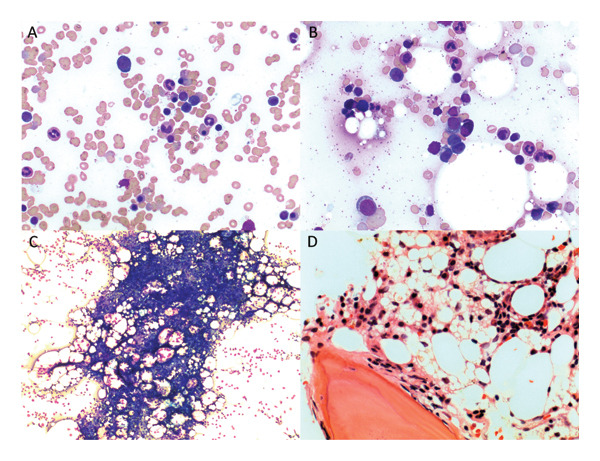
Bone marrow aspirate and biopsy. (A, B) Bone marrow aspirate 500x magnification, Wright–Giemsa stain, showing hematopoiesis in different regions. (C) Bone marrow aspirate 40x magnification, Wright–Giemsa stain, showing a marrow particle with absence of megakaryocytes. (D) Bone marrow core biopsy 100x magnification, hematoxylin and eosin stain, showing cross section of marrow core with absence of megakaryocytes.

Bone marrow aspirate and biopsy performed a month later revealed an absence of MKs (Figure 1). Aspirate morphology demonstrated normal granulopoiesis and erythropoiesis without an increase in blasts or evidence of atypical lymphoid populations. Cytogenetics revealed loss of the Y chromosome in 8 of the 19 cells analyzed (42.1%), favored to be secondary to a normal age‐related event. The bone marrow biopsy also showed a complete absence of MKs, no amyloid deposition on Congo‐red stain, and no evidence of a lymphoplasmacytic neoplasm of significance.

On imaging, computerized tomography scan of the chest, abdomen, and pelvis did not show any evidence of lymphadenopathy, organomegaly or other evidence of malignancy. No specific MK cutoff is widely used for the diagnosis of AAMT, and as such, given the absence of MKs, normal erythropoiesis, and exclusion of other causes, a diagnosis of AAMT was made. However, a diagnosis of AA could not initially be made as the bone marrow biopsy did not demonstrate trilineage hypocellularity of < 25%; no features of severe AA (i.e., absolute neutrophil count < 0.5, platelets < 20, and reticulocytes < 60), as per the Camitta criteria, were present.

The presence of a PNH clone reinforced our diagnosis of AAMT as PNH clones are often associated with immune‐mediated bone marrow failure syndromes. Given that the patient did not initially satisfy the diagnostic criteria for AA, had no evidence of thrombosis, but did have absent MKs with preserved hematopoiesis in other cell lineages, the PNH clone helped us to further make the diagnosis of AAMT as opposed to AA or classical PNH.

His allopurinol was discontinued due to its risk of cytopenias. He received a 4‐day course of dexamethasone 40 mg once daily with no improvement in his platelet counts. Subsequently, a month after, he was prescribed eltrombopag 50 mg once daily, cyclosporine 200 mg twice daily, and prednisone 1 mg/kg (80 mg) once daily. Eltrombopag was titrated to 150 mg once daily after approximately 3 weeks. Cyclosporine was titrated gradually aiming for a cyclosporine trough level of 150–250 ng/mL. The patient was monitored closely with bloodwork every 1‐2 weeks initially and with dose adjustments and subsequently, the frequency of clinic visits and bloodwork was reduced. Cyclosporine continued for over 1 month but discontinued due to renal and hepatotoxicity, with a rise in creatinine from 101 μmol/L to 150 μmol/L, alanine aminotransferase from 26 to 88 U/L (reference range < 41), and GGT from 29 to 147 U/L. The cyclosporine trough level after approximately 1 month of therapy was 312 ng/mL. Prednisone was tapered rapidly and discontinued over a month due to adverse effects, including shakiness and decreased appetite. Specifically, the patient was on 80 mg once daily for 2 weeks and 50 mg once daily for 2 weeks until prednisone was discontinued altogether. Given the patient’s age and comorbidities, he was deemed too frail for more aggressive therapies, such as plasmapheresis, antithymocyte globulin (ATG), or stem cell transplant. The goal was to achieve and maintain a platelet count above 30 × 10^9^/L for daily activities and 50 × 10^9^/L and above for resuming anticoagulation and/or undergoing surgical procedures in the future. The patient did not receive any platelet transfusions or intravenous immunoglobulin (IVIg) prior to eltrombopag therapy. He did not require hospitalization.

**FIGURE 2:**
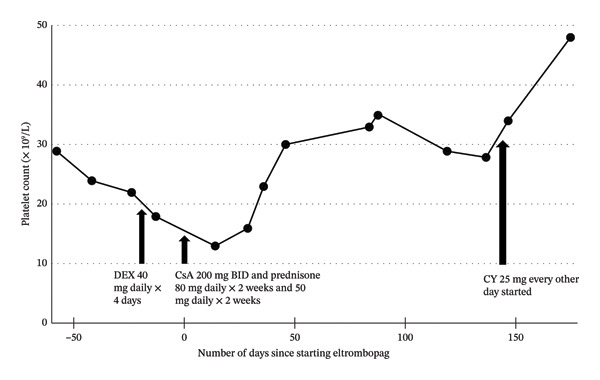
Changes in platelet count over the course of treatment with eltrombopag, dexamethasone (DEX), cyclosporine (CsA), prednisone, and cyclophosphamide (CY); BID = twice daily.

Ultimately, the patient continued eltrombopag 150 mg once daily with improved platelet counts, 48 × 10^9^/L 6 months after treatment compared with a nadir of 18 × 10^9^/L prior (Figure 2). In addition, he was started on cyclophosphamide 25 mg every other day. The platelets rose by 14 × 10^9^/L 1 month after cyclophosphamide was added. Over time, his bruising also improved and he was able to resume his edoxaban and digoxin as his platelet count was above 50 × 10^9^/L [11]. Throughout this treatment period, he did not require any platelet transfusions. He did, however, require two units of packed red blood cells for his hemolytic anemia that was attributed to his PNH clone and potential ineffective hematopoiesis; his hemoglobin rose from 73 to 98 g/L after the transfusion. Complete blood counts and liver function tests were monitored every month during therapy.

For thrombosis risk mitigation, we ensured to carefully titrate the dose of eltrombopag with close monitoring of platelet counts. The patient was monitored regularly for clinical signs and symptoms of thrombosis. Furthermore, we assessed the patient for additional thrombotic risk factors, including the presence of a PNH clone. To minimize the risk of hepatotoxicity, the patient’s liver function tests were assessed once every 1‐2 weeks during dose increases of eltrombopag. Furthermore, the patient was monitored closely for visual changes to assess for cataract formation. He did not have any adverse drug effects (ADEs) from eltrombopag, with no bleeding, thrombosis, cataract development, hepatotoxicity, or cytopenias. The patient was instructed to take eltrombopag on an empty stomach with separation from polyvalent cations, including calcium, magnesium, iron, or aluminum containing products, to increase bioavailability and minimize chelation.

Ultimately, his hemolytic markers stabilized with eltrombopag. Specifically, his LDH peaked at 595 U/L 1 month into treatment and later fell to 468 U/L 6 months into treatment. His total and unconjugated bilirubin peaked at 60 and 25 μmol/L, respectively, 1 month into treatment and later fell to 19 and 9 μmol/L, respectively, 6 months into treatment. His reticulocytes peaked at 111 × 10^9^/L 1 month into treatment and later fell to 97 × 10^9^/L 3 months into treatment. After 3 months of treatment with eltrombopag, the clone size of GPI deficient neutrophils fell from 0.79% to 0.2%; the clone size of total GPI deficient red blood cells fell from 0.47% to 0.23%. His macrocytosis initially increased from an initial MCV of from 114.6 fL to a peak of 121.8 fL after 10 months of treatment with eltrombopag but later improved to 107.3 fL 18 months into treatment.

It is important to emphasize that cyclosporine and cyclophosphamide were given concurrently with eltrombopag and, as such, are confounding factors. Therefore, the relationship between the patient’s platelet recovery and eltrombopag is best described as a temporal association rather than a direct causal effect.

Interestingly, his repeat bone marrow aspiration and biopsy, which was performed 3 months into treatment with eltrombopag, showed a potential evolving AA as there was trilineage hypocellularity, which was not present in the initial bone marrow findings. A summary of the chronological timeline of the case description is presented in Table A1.

## 3. Discussion

AAMT is a rare disorder characterized by reduced or absent MKs in the absence of other hematological abnormalities [2]. Given that there have been less than a hundred cases of AAMT reported in the literature, there is limited information available regarding treatments for AAMT. To date, there have been only 15 patient cases published in the literature in which TPO‐RAs were used (Table A2). Our case demonstrates that eltrombopag can be effective in both increasing platelet levels and decreasing bleeding in patients with AAMT. Specifically, our patient had a 30‐point increase in his platelet levels after 6 months of treatment with eltrombopag. Additionally, there were no adverse outcomes observed with our patient case after eltrombopag administration, including no bleeding, thrombosis, or death.

Several studies have reported similar findings. Cela et al. reported on a patient with AAMT and systemic lupus erythematosus whose platelets rose from 22 × 10^9^/L to 179 × 10^9^/L after 6 weeks with eltrombopag 50 mg daily. The patient had failed on glucocorticoids, rituximab, and cyclosporine [12]. Suyama et al. studied a patient whose platelets rose from 7 × 10^9^/L to 223 × 10^9^/L after 86 days with a tapering course of eltrombopag (maximum dose 50 mg daily); glucocorticoids also proved to be ineffective in this patient [17]. Likewise, Anwar et al. published a case report where the patient’s platelets increased to 35 × 10^9^/L from < 5 × 10^9^/L with an unknown duration of eltrombopag 75 mg daily. There was no significant platelet increase with IVIg or steroids [3]. Interestingly, Hussain et al. demonstrated that eltrombopag can even be effective when another TPO‐RA is insufficient as their patient case had at least a 10‐point platelet increase after 6 months of eltrombopag (maximum dose 125 mg daily) but did not have a significant rise in platelets with 13 weeks of romiplostim, IVIg, steroids, rituximab, cyclosporine, or azathioprine [18]. There were no ADEs associated with eltrombopag in any of these aforementioned studies. These studies echo our findings of improved platelet counts in our patient case after treatment with eltrombopag without the development of any ADEs.

Other TPO‐RAs have also proven to be useful in treating AAMT. Tu et al. published two cases where there were no significant changes in platelet numbers with eltrombopag, recombinant TPO (rh‐TPO), IVIg, glucocorticoids, cyclosporine, or granulocyte colony–stimulating factor (G‐CSF). However, both patients’ platelets increased by > 90 points after 3 months of treatment with avatrombopag 40 mg daily [18]. Furthermore, Shigekiyo et al. reported on a patient whose platelets increased from 4 × 10^9^/L to 1002 × 10^9^/L after 1 year of treatment with romiplostim (maximum dose 3–5 mcg/kg/week). The patient previously had not responded significantly to eltrombopag, glucocorticoids, cyclosporine, ATG, cyclophosphamide, or rituximab [4]. Additionally, a case report by Zimmerman et al. highlighted that a 1‐year course of romiplostim (1 mcg/kg/week) resulted in a 75–85 platelet increase in a pregnant patient after she had not responded to glucocorticoids or IVIg [16]. There were no ADEs associated with either avatrombopag or romiplostim in these studies.

Our patient case highlighted the synergistic effects of eltrombopag when combined with other medications, namely, cyclosporine and cyclophosphamide. Interestingly, several studies have demonstrated improved platelet counts in AAMT with TPO‐RAs combined with other medications. Tian et al. reported on two cases where the platelet count rose from < 10 × 10^9^/L to the reference range after 12 weeks with eltrombopag and cyclosporine in the first case and 4 weeks of eltrombopag and cyclosporine (later changed to tacrolimus due to gingival hyperplasia) in the second case. In both cases, the platelet counts remained in the reference range even a year after eltrombopag was discontinued. These patients had also failed treatment with IVIg, rh‐TPO, glucocorticoids, and rituximab [19]. Anwar et al. published a second case in which there was no significant increase in platelets with eltrombopag alone; however, when cyclosporine was added, the platelets rose from 8.35 × 10^9^/L to 16 × 10^9^/L after several weeks. The platelets eventually reached the reference range, but cyclosporine had to be discontinued after a couple months due to GI ADEs [3]. Moreover, Pardal de la Mano et al. reported on a patient in which there was no significant increase in platelet numbers with eltrombopag, but when cyclosporine was added, the platelets rose from < 10 × 10^9^/L to 200 × 10^9^/L over a course of 450 days. Importantly, the platelets dropped back to < 10 × 10^9^/L after cyclosporine was discontinued and the dose of eltrombopag was reduced. Ultimately, the platelets increased to 150 × 10^9^/L when cyclosporine was added again and the dose of eltrombopag was increased. The platelets remained at approximately 125 × 10^9^/L even after eltrombopag was discontinued, which illustrates the importance of both eltrombopag and cyclosporine in this patient’s management [15]. Similar findings have been demonstrated with avatrombopag as well. Specifically, Meng et al. published a patient case where the platelets increased from < 60 × 10^9^/L to 175 × 10^9^/L after 20 days of treatment with avatrombopag and cyclosporine. The patient had previously failed treatment with TPO‐RA monotherapy, including both eltrombopag and hetrombopag, along with a number of other therapies outlined in Table A2 [21]. No TPO‐RA ADEs were observed in any of these cases. Interestingly, our patient’s platelets rose by 14 × 10^9^/L after cyclophosphamide was added to eltrombopag. Cyclophosphamide may have contributed to the clinical efficacy of eltrombopag given that it specifically targets B‐lymphocytes, which is important in the context of the IgG lambda peak on SPEP. However, his IgG lambda peak did not reduce after 6 months of cyclophosphamide therapy but remained stable at 2.6 g/L. Ultimately, there is a lack of literature on the synergistic effects of cyclophosphamide and TPO‐RAs, making it an important topic for future investigation.

All of the aforementioned cases suggest the potential effectiveness of TPO‐RAs, either alone or combined with other medications, in treating AAMT. TPO‐RAs exert their effect on MKs through activation of key signaling pathways, such as the Janus kinase/signal transducer and activator of transcription (JAK‐STAT), mitogen‐activated protein kinase (MAPK), and phosphatidylinositol 3‐kinase (PI3K‐AKT). Activation of these pathways results in stem cell stimulation, MK proliferation, and prevention of platelet apoptosis [22]. However, Novotny et al. reported on a patient who did not have a sustained rise in platelets with eltrombopag, romiplostim, or cyclosporine. The platelets ultimately dropped to < 50 × 10^9^/L despite several weeks of therapy. The patient then developed pancytopenia secondary to AA, and allogenic stem cell transplantation was considered as a last line option [13]. Several other studies have reported on patient cases of AAMT progressing to AA, which may suggest that AAMT potentially results from an acquired stem cell defect and thus is on a spectrum with AA [14, 18, 20, 23–27]. Specifically, severe thrombocytopenia with macrocytic anemia are two features of the early stages of AA [28]; both of these findings were present in our case of AAMT as well. Furthermore, the repeat bone marrow aspiration and biopsy for our patient case demonstrated new trilineage hypocellularity, suggesting likely progression to AA. This observation supports prior aforementioned reports proposing that AAMT and AA may represent a disease spectrum characterized by immune‐mediated bone marrow failure, in which early selective suppression of megakaryopoiesis may precede the development of broader hematopoietic failure. Interestingly, our patient case had a PNH clone, which has been found in other cases of AAMT [16, 29]. PNH clones have also been found in 60% of the AA patients and 20% of the patients with low‐risk MDS [28], supporting the concept that immune‐mediated selective pressure within the bone marrow may permit the expansion of PNH hematopoietic clones. Our group reported another AAMT patient with PNH clones and speculated whether autoimmunity plays a role in the pathophysiology of AAMT [29]. Taken together, these findings further suggest that AAMT, AA, and PNH may share overlapping immune‐mediated mechanisms within the spectrum of bone marrow failure syndromes. However, the precise clinical significance of PNH clones in AAMT remains unclear and warrants further investigation. Likewise, further research is required to delineate the exact pathophysiology linking AAMT and AA. Ultimately, eltrombopag has been demonstrated to work well in both AA and AAMT, which is likely due to its proliferative effect on both hematopoietic stem cells and MKs, making it effective in treating bone marrow failure syndromes [7–9].

Overall, including our case, there are 16 cases of AAMT treated with TPO‐RAs. Specifically, there are 15 cases treated with eltrombopag, three with avatrombopag, four with romiplostim, and one with hetrombopag. Responses were reported in 94% of the published cases; however, this estimate is derived primarily from case reports and small case series and may overestimate the true efficacy due to publication and reporting bias. With eltrombopag, doses used ranged from 12.5 mg up to 150 mg. Responses in patients on eltrombopag appear better in those with higher daily doses than those with lower daily doses. However, while our case suggests possible benefit of TPO‐RAs, it is difficult to fully evaluate the effectiveness of TPO‐RAs for two main reasons. First, TPO‐RA treatment tends to be long term and the presence of other short‐term immunosuppressive therapies and spontaneous remission/variation can confound the results. Additionally, reporting bias may lead to an overestimation of TPO‐RA efficacy. Nonetheless, our case contains several novel features, including a very elderly patient, presence of a PNH clone, response after cyclosporine cessation, and emerging AA features.

## 4. Conclusion

AAMT is a rare bleeding disorder with less than a hundred cases reported in the literature and 15 cases treated with TPO‐RAs. Although not fully understood, AAMT is also a disorder with heterogenous etiologies and overlaps with bone marrow failure syndromes and autoimmune diseases as we have described. Our case and those reported in the literature demonstrate variability in the management of AAMT. TPO‐RAs appear to be effective in patients with AAMT but responses are variable and patients appear to respond to one TPO‐RA even if they have failed to respond to another TPO‐RA. Furthermore, the addition of therapies such as cyclosporine may augment the effect of TPO‐RAs in patients with AAMT. Future research directions include comparing the effectiveness of different TPO‐RAs, evaluating the optimal dosage and duration of TPO‐RA therapy, examining pertinent ADEs of TPO‐RAs, and assessing synergistic effects between TPO‐RAs and other medications. Moreover, it would be worthwhile to further examine the role of autoimmunity in AAMT by measuring serum anti‐c‐Mpl antibodies and TPO levels; the serum TPO level in particular would aid in elucidating the etiology of AAMT and assessing the effectiveness of TPO‐RAs. Lastly, the link between AA and AAMT requires further investigation. Although it is difficult to differentiate AAMT from an evolving AA in our patient case, we nonetheless demonstrate that TPO‐RAs can be effective novel therapeutic agents and improve patient outcomes for these rare bone marrow failure syndromes.

## Funding

This research did not receive any specific grant from funding agencies in the public, commercial, or not‐for‐profit sectors.

## Ethics Statement

Ethics approval was not required for the publication of this manuscript.

## Consent

The patient provided informed written consent for this manuscript to be published.

## Conflicts of Interest

The authors declare no conflicts of interest.

## Data Availability

The data that support the findings of this study are available from the corresponding author upon reasonable request.

## Appendix A

**TABLE A1:** Summary of chronological events of case description.

Presenting symptoms	Platelet counts (× 10^9^/L)	Absolute neutrophil count (ANC) (× 10^9^/L)	Hemoglobin (g/L)	Transfusions	Treatments	Clinical events
‐ Fatigue ‐ Petechiae ‐ Purpura ‐ Ecchymoses	‐ Initial: 29 ‐ Nadir (< 50 days after treatment with eltrombopag): 18 ‐ 125 days after eltrombopag added: 28 ‐ 6 months after treatment with eltrombopag and 1 month after cyclosporine added: 48	‐ Initial: 2.5 ‐ 6 months after treatment with eltrombopag: 3.0	‐ Initial: 105 ‐ 4 months after treatment with eltrombopag: 73 ‐ Post‐transfusion: 98 ‐ 6 months after treatment with eltrombopag: 93	‐ 2 units of packed red blood cells given approximately 4 months after treatment with eltrombopag (hemoglobin count rose from 73 to 98)	‐ Dexamethasone 40 mg oral once daily: 4 days ‐ Cyclosporine 200 mg oral every 12 h: 1 month ‐ Prednisone 80 mg oral once daily: 2 weeks ‐ Prednisone 50 mg oral once daily: 2 weeks ‐ Cyclophosphamide 25 mg oral every other day: added 5 months after treatment with eltrombopag	‐ 6 months after treatment with eltrombopag: fatigue, petechiae, purpura, and ecchymoses all improved ‐ No bleeding or thrombosis

**TABLE A2:** Summary of case reports of AAMT treated with TPO‐RAs.

Study	Number of cases	Patient age and sex	Therapy	Other therapies used	Outcome
Cela et al. [12]	1	55 F	‐ Eltrombopag 50 mg daily × at least 6 weeks	‐ Prednisone 70 mg daily × 3 weeks, rituximab weekly (dose not known) × 2 months, cyclosporine 150 mg daily × 1 week	‐ Platelets rose from 22 to 179 after 6 weeks with eltrombopag ‐ No bleeding, thrombosis, or death
Shigekiyo et al. [4]	1	55 M	‐ Eltrombopag 12.5 mg/day (dose increased by 12.5 mg/day every 2 weeks); 50 mg/day × 1 month ‐ Romiplostim 1 mcg/kg once weekly (gradually increased to 3‐5 mcg/kg). Taken for at least 1 year	‐ Methylprednisolone 1 g/day × 3 days, prednisolone 1 mg/kg/day × 1 month, cyclosporine 5 mg/kg/day × 2 months, Horse ATG 1 g/day × 5 days, Rabbit ATG 0.5 g/day × 5 days, danazol 300 mg/day × 3 months, cyclophosphamide 1 g + 2 g, rituximab 375 mg/m^2^ × 2 doses	‐ No increase in platelet count with eltrombopag. ‐ Initial platelet count 4; platelets 1002 after 1 year with romiplostim. ‐ No bleeding, thrombosis, or death
Novotny et al. [13]	1	61 M	‐ Eltrombopag 50 mg daily × several weeks, then 75 mg daily × several weeks ‐ Romiplostim (dose unknown)	‐ Prednisone 250 mg/day (duration unknown), dexamethasone 40 mg/day × 4 days, IVIg 80 g + 110 g, cyclosporine (dose unknown)	‐ No sustained increase in platelet count with eltrombopag, romiplostim, or cyclosporine. Platelets < 50. ‐ No bleeding, thrombosis, or death. Aplastic anemia developed.
^∗^Levy et al. [14]	1	Not reported	Eltrombopag	Cyclosporine, ATG	“Complete recovery” as based on abstract
Pardal de la Mano et al. [15]	1	58 M	‐ Eltrombopag 50 mg/day (subsequently increased to 100 mg/day; dose then decreased and increased to unknown amounts) × ∼2 years	‐ IVIg 30 g/day × 5 days, prednisone 90 mg/day (duration unknown) azathioprine × 1 month (dose unknown), cyclosporine 300 mg/day × ∼3 years	‐ No improvement in platelet count with eltrombopag + prednisone after 2 months, but petechiae disappeared. ‐ Platelets increased from ∼ 0 to 200 after 450 days with eltrombopag + cyclosporine, then decreased to ∼ 0 again after cyclosporine was discontinued + eltrombopag dose reduced, then increased to 150 over 450 days after eltrombopag dose increased + cyclosporine added, and remained ∼125 after eltrombopag discontinued. ‐ No bleeding, thrombosis, or death
Zimmerman et al. [16]	1	35 F (22 weeks’ gestation)	Romiplostim 10 mcg/kg/week for at least 1 year	Prednisone, IVIg (Doses unknown)	‐ Platelets increased from 5 to 80–90 after 1 year with romiplostim ‐ No bleeding, thrombosis, or death.
Suyama et al. [17]	1	79 M	‐ Eltrombopag 25 mg daily 1 month, 50 mg daily × 1 month, 37.5 mg daily × 2 weeks, 25 mg daily × 1 week, 12.5 daily × 1 week	‐ Prednisolone 50 mg/day taper for at least 13 days	‐ Platelet count increased from 7 to 223 after 86 days with eltrombopag ‐ No bleeding, thrombosis, or death related to TPO‐RA.
Tu et al. [18]	2	67 M 71 F	67 M: eltrombopag 50 mg daily × 3 months, avatrombopag 40 mg daily (taken for at least 3 months) 71 F: eltrombopag 50 mg daily × 2 weeks, avatrombopag 40 mg daily (taken for at least 2 months)	67 M: rh‐TPO 15 000 units daily × 13 days, methylprednisolone 80 mg daily × 3 weeks, IVIg 20 g daily × 5 days, cyclosporine 100 mg daily × 1 week 71 F: rh‐TPO 15 000 units daily × 6 weeks, IVIg 20 g daily × 5 days, methylprednisolone 40 mg daily × 2 weeks, G‐CSF 200 units daily × 2 weeks	67 M: ‐ No increase in platelet count with eltrombopag. ‐ Platelet count rose from 5 to 100 after 2 months with avatrombopag. ‐ No bleeding, thrombosis, or death 71 F: ‐ No increase in platelet count with eltrombopag. ‐ Platelet count rose from 2 to 94 after 2 months with avatrombopag. ‐ No bleeding, thrombosis, or death
Tian et al. [19]	2	15 M 16 M	15 M: ‐ Eltrombopag 50 mg/day × 8 months 16 M: ‐ Eltrombopag 75 mg/day (titrated up to 150 mg/day) × 2 years	15 M: IVIg 70 g/day × 2 days, rh‐TPO 300 μ/kg/day × ∼ 2 days, methylprednisolone 40 mg/day × ∼ 60 days, cyclosporine 3 mg/kg/day × ∼ 1 year 16 M: IVIg (dose + duration unknown), rh‐TPO 300 μ/kg/day × 14 days, methylprednisolone 80 mg/day × ∼ 100 days, rituximab 375 mg/m^2^ × 4 infusions, cyclosporine 3 mg/kg/day × ∼ 50 days, tacrolimus (dose + duration unknown)	15 M: ‐ Platelet count rose from 1 to 200 after 12 weeks with eltrombopag and cyclosporine. ‐ Platelets remained in the reference range 18 months after eltrombopag and cyclosporine were discontinued. ‐ No bleeding, thrombosis, or death 16 F: ‐ Platelet count rose from 5 to reference range after 4 weeks with eltrombopag 150 mg + cyclosporine (changed to tacrolimus). Eltrombopag withdrawn after 2 years. Platelets still in the reference range after 36 months of follow‐up. ‐ No bleeding, thrombosis, or death
Hussain et al. [20]	1	71 F	‐ Romiplostim 1 mcg/kg/week (titrated up to 10 mcg/kg/week) × 13 weeks ‐ Eltrombopag 50 mg once daily (titrated up to 125 mg once daily) × 6 months	‐ IVIg (dose unknown), prednisone 1 mg/kg × 1 week, rituximab 375 mg/m^2^ weekly × 4 doses, cyclosporine 2 mg/kg BID (target trough level 150–400 ng/mL) × 3 months, azathioprine 200 mg once daily × 1 month	‐ No sustained increase in platelet count with romiplostim. Developed aplastic anemia. ‐ Platelets increased from < 20 to 30 after 6 months with eltrombopag ‐ No bleeding, thrombosis, or death.
Anwar et al. [3]	2	50 M 51 F	50 M: ‐ Eltrombopag 50 mg once daily (titrated up to 75 mg once daily). Duration unknown. 51 F: ‐ Eltrombopag 100 mg once daily (titrated down to 50 mg once daily; duration unknown)	50 M: IVIg, steroids (doses and duration unknown) 51 F: IVIg, steroids, cyclosporine (doses and duration unknown)	50 M: ‐ Platelets increased to 35 from < 5 with eltrombopag ‐ No bleeding, thrombosis, or death 51 F: ‐ No increase in platelet count with eltrombopag. ‐ Platelets rose to 16 from 8.35 several weeks after cyclosporine added to eltrombopag. Cyclosporine discontinued after few months due to GI ADE. Platelets normalized ‐ No bleeding, thrombosis, or death
Meng et al. [21]	1	44 F	‐ Eltrombopag 50 mg daily × 80 days, hetrombopag 7.5 mg daily × 18 days, avatrombopag 60 mg daily for at least 2.5 months	‐ Rh‐TPO 15 000 units daily × 98 days, G‐CSF 5 mcg/kg/day × 18 days, testosterone undecanoate 120 mg/day × 80 days, IVIg 20 g/day × 6 days, dexamethasone 10 mg daily (unknown duration), cyclosporine 20 mg daily (dose adjusted to keep trough level between 100 and 200 ng/mL) for at least 2.5 months	‐ No sustained increase in platelet count with eltrombopag or hetrombopag. ‐ Platelets increased from 15 to 60 to 175 with 20 days of avatrombopag and cyclosporine ‐ No bleeding, thrombosis, or death

*Note:* All platelet counts reported as × 10^9^/L. TPO‐RA = thrombopoietin receptor agonist; rh‐TPO = recombinant TPO; IVIg = intravenous immunoglobulin; BID = twice daily.

Abbreviations: ADEs, adverse drug effects; ATG, antithymocyte globulin; G‐CSF, granulocyte colony–stimulating factor.

^∗^Unable to access full article. Information in table based on the abstract.
